# Combined assessment of fibrinogen-to-platelet ratio and ApoB/ApoA1 ratio for pre-angiographic risk stratification of severe multivessel coronary disease in NSTEMI

**DOI:** 10.3389/fcvm.2026.1931950

**Published:** 2026-10-05

**Authors:** Zongqi Zhou, Wei Wang, Haoyu Zhou, Zhiliang Chen, Mingming Jiang, Zimeng Li, Lei Li

**Affiliations:** 1 Department of General Practice, The Affiliated Hospital of Xuzhou Medical University, Xuzhou, China; 2 Department of Nephrology, The Affiliated Hospital of Xuzhou Medical University, Xuzhou, China; 3 Department of Statistics, Columbia University, New York, NY, United States

**Keywords:** ApoB/ApoA1 ratio, FPR, MVD, nomogram, NSTEMI

## Abstract

**Background:**

Residual inflammation and dysregulated lipid metabolism are closely associated with the extent of severe multivessel coronary disease (MVD) in patients with non-ST-segment elevation myocardial infarction (NSTEMI). The fibrinogen-to-platelet ratio (FPR) and the apolipoprotein B/apolipoprotein A1 (ApoB/ApoA1) ratio reflect these two residual risk pathways, respectively, whereas the clinical value of their combined assessment for pre-angiographic identification of severe MVD remains to be evaluated.

**Objective:**

To evaluate the incremental value of combining FPR with the ApoB/ApoA1 ratio for pre-angiographic identification of severe MVD in patients with NSTEMI and to develop an adjunctive risk-stratification model.

**Methods:**

This single-center retrospective study included 580 patients with NSTEMI who underwent coronary angiography. Correlation analyses, logistic regression, and restricted cubic spline models were used to assess the associations between the two biomarkers and MVD. The area under the receiver operating characteristic curve (AUC), continuous net reclassification improvement (NRI), and integrated discrimination improvement (IDI) were used to evaluate the incremental value of the biomarkers. A nomogram was developed and internally validated using bootstrap resampling. Decision curve analysis (DCA) was performed to assess net clinical benefit, and subgroup analyses were conducted.

**Results:**

FPR remained independently and positively associated with severe MVD, with an approximately linear dose-response relationship; the association of the ApoB/ApoA1 ratio was attenuated after full adjustment, although it showed complementary value in patients with a very high coronary lesion burden. With the combined model, the AUC increased from 0.683 to 0.719, while the continuous NRI and IDI were 0.488 and 0.037, respectively; adding the ApoB/ApoA1 ratio to the model containing FPR did not significantly improve the AUC (*P* = 0.266) but yielded further improvements in NRI and IDI. Potential effect modification by diabetes status was observed (*P* for interaction = 0.025).

**Conclusion:**

FPR is an important adjunctive biomarker for the pre-angiographic identification of severe MVD in patients with NSTEMI, whereas the ApoB/ApoA1 ratio provides complementary reclassification information. The nomogram incorporating both biomarkers may facilitate pre-angiographic risk stratification.

## Highlights

- For the first time, residual inflammatory (FPR) and lipid (ApoB/ApoA1) risk pathways were jointly assessed to support pre-angiographic MVD stratification in patients with NSTEMI.
- Diabetes status may attenuate the association between the two biomarkers and severe MVD (*P* for interaction = 0.025), suggesting that dysregulated glucose metabolism may influence the risk information conveyed by the inflammation–lipid axis and providing a rationale for stratified risk assessment;
- The model relies primarily on routine clinical and laboratory variables available at admission, demonstrates good internal calibration and modest additional net clinical benefit, and may assist with the pre-angiographic identification of complex coronary lesions and clinical management planning in patients with NSTEMI.

## 1 Introduction

Non-ST-segment elevation myocardial infarction (NSTEMI) accounts for approximately 70% of all hospitalized patients with acute coronary syndrome (ACS) (1). It has become the predominant clinical subtype of acute myocardial infarction worldwide (2), with its incidence continuing to rise (3, 4). Compared with ST-segment elevation myocardial infarction patients, those with NSTEMI often exhibit a more extensive burden of coronary atherosclerosis; diagnostic coronary angiography identifies multivessel coronary artery disease (MVD) in 30%–70% of NSTEMI admissions (5). MVD is a well-established poor prognostic marker linked to incident heart failure, recurrent ischemic events, and long-term all-cause mortality (6). Furthermore, MVD status directly guides clinical decisions regarding revascularization modalities, including percutaneous coronary intervention (PCI) and coronary artery bypass grafting (CABG) (7, 8). Accordingly, reliable noninvasive identification of severe MVD prior to invasive coronary angiography is essential to refine risk stratification and formulate individualized revascularization strategies (9).

Despite standard lipid-lowering, antiplatelet, and revascularization therapies currently recommended by guidelines, a substantial proportion of patients with NSTEMI remain at high risk for the progression of atherosclerotic lesions and recurrent cardiovascular events (10), the core trigger of which is residual cardiovascular risk (11). Residual inflammatory risk and residual lipid risk represent two complementary pathological pathways that jointly mediate endothelial dysfunction, plaque progression, and the development of complex coronary lesions (12). The excessive activation of chronic inflammation accelerates thrombosis and promotes plaque destabilization (13); meanwhile, even when low-density lipoprotein cholesterol reaches therapeutic targets, the continuous accumulation of pro-atherogenic lipoproteins still causes widespread vascular lipid deposition (14). Multiple studies have demonstrated that persistent interactions between inflammatory and lipid pathways are closely associated with the development and progression of extensive multivessel coronary atherosclerosis (15). Against this background, there is an urgent clinical need for biomarkers capable of simultaneously quantifying residual inflammatory and lipid risks to comprehensively assess the coronary atherosclerotic burden.

The fibrinogen-to-platelet ratio (FPR), integrating circulating fibrinogen and platelet counts, reflects the activation state of the inflammation-coagulation axis and serves as a surrogate marker for residual inflammatory risk (16). Conversely, the apolipoprotein B/apolipoprotein A1 (ApoB/ApoA1) ratio quantifies the balance between the total burden of pro-atherogenic lipoproteins and the function of protective high-density lipoproteins, representing the degree of residual systemic lipid metabolic disturbance (17, 18). Previous cohort studies have demonstrated that when used individually, both indicators are associated with the severity of coronary lesions and adverse cardiovascular events (19, 20). Traditional risk assessment tools commonly used in clinical practice have important limitations in specific clinical applications. For example, the GRACE risk score primarily focuses on hard clinical endpoints such as mortality and relies on multiple clinical variables available at admission, whereas the SYNTAX score depends entirely on the anatomical disease burden assessed by invasive coronary angiography (21, 22). There remains a lack of noninvasive risk assessment tools based on routine and readily available biochemical markers that can be used before coronary angiography. Most previous studies have examined the FPR or the ApoB/ApoA1 ratio individually; to date, no clinical study has combined the two biomarkers for noninvasive pre-angiographic identification of severe MVD in patients with NSTEMI. Although close pathological interactions exist between residual inflammatory and lipid pathways during atherosclerotic progression, no study has yet developed a diagnostic nomogram integrating these dual-pathway indicators, nor has any study explored whether the association between the combined biomarkers and severe MVD varies according to metabolic status, particularly diabetes.

Considering the current research landscape, this study adopted a single-center, retrospective cross-sectional design and consecutively enrolled hospitalized patients with NSTEMI who underwent invasive coronary angiography between February 2024 and May 2026. We hypothesized that the combined assessment of FPR and the ApoB/ApoA1 ratio to assess residual inflammatory and lipid risks could independently aid in identifying MVD, and that the association between the combined biomarkers and MVD would vary according to diabetes status. The primary objectives of this study were to analyze the linear dose-response relationships between the two biomarkers and MVD risk; quantify the incremental clinical value of combining the two biomarkers for discriminating MVD; and construct and validate a nomogram to provide an adjunctive, noninvasive tool for pre-angiographic risk stratification in patients with NSTEMI scheduled to undergo coronary angiography (23).

## 2 Methods

### 2.1 Study design and population

This was a single-center, retrospective cross-sectional study that consecutively enrolled patients with NSTEMI who were hospitalized and underwent coronary angiography (CAG) at the Affiliated Hospital of Xuzhou Medical University between February 2024 and May 2026. Given that all participants were hospitalized patients who had undergone invasive coronary angiographic evaluation, the findings of this study are primarily applicable to moderate- to high-risk patients with NSTEMI in whom there is a high clinical suspicion of coronary artery disease. The diagnosis of NSTEMI was established in accordance with the 2025 American College of Cardiology/American Heart Association (ACC/AHA) guidelines for the management of ACS (1). Detailed inclusion and exclusion criteria were defined (Table 1).

**Table 1:** Inclusion and exclusion criteria of the study population.

Category	Selection criteria
Inclusion criteria	(1) age ≥ 18 years (2) clinically confirmed diagnosis of NSTEMI (3) completion of CAG during hospitalization (4) availability of complete clinical and laboratory data prior to CAG
Exclusion criteria	(1) severe hepatic or renal dysfunction (2) active infection, sepsis, or autoimmune diseases (3) malignant tumors or severe malnutrition (4) primary hematological diseases or recent major bleeding events (5) a history of PCI or CABG, or concurrent severe valvular heart disease, congenital heart disease, or primary cardiomyopathy (6) missing key clinical or laboratory data

The inclusion criteria were as follows: (1) age ≥ 18 years; (2) clinically confirmed diagnosis of NSTEMI; (3) completion of CAG during hospitalization; (4) availability of complete clinical and laboratory data prior to CAG.

The exclusion criteria were as follows: (1) severe hepatic or renal dysfunction; (2) active infection, sepsis, or autoimmune diseases; (3) malignant tumors or severe malnutrition; (4) primary hematological diseases or recent major bleeding events; (5) a history of PCI or CABG, or concurrent severe valvular heart disease, congenital heart disease, or primary cardiomyopathy; (6) missing key clinical or laboratory data.

1,593 patients with NSTEMI were screened. After excluding 1,013 patients based on the aforementioned criteria, a final total of 580 patients were included in the analysis. Based on the CAG results, they were divided into a non-MVD group (*n* = 189) and an MVD group (*n* = 391). The case screening flowchart is depicted in Figure 1.

**Figure 1:**
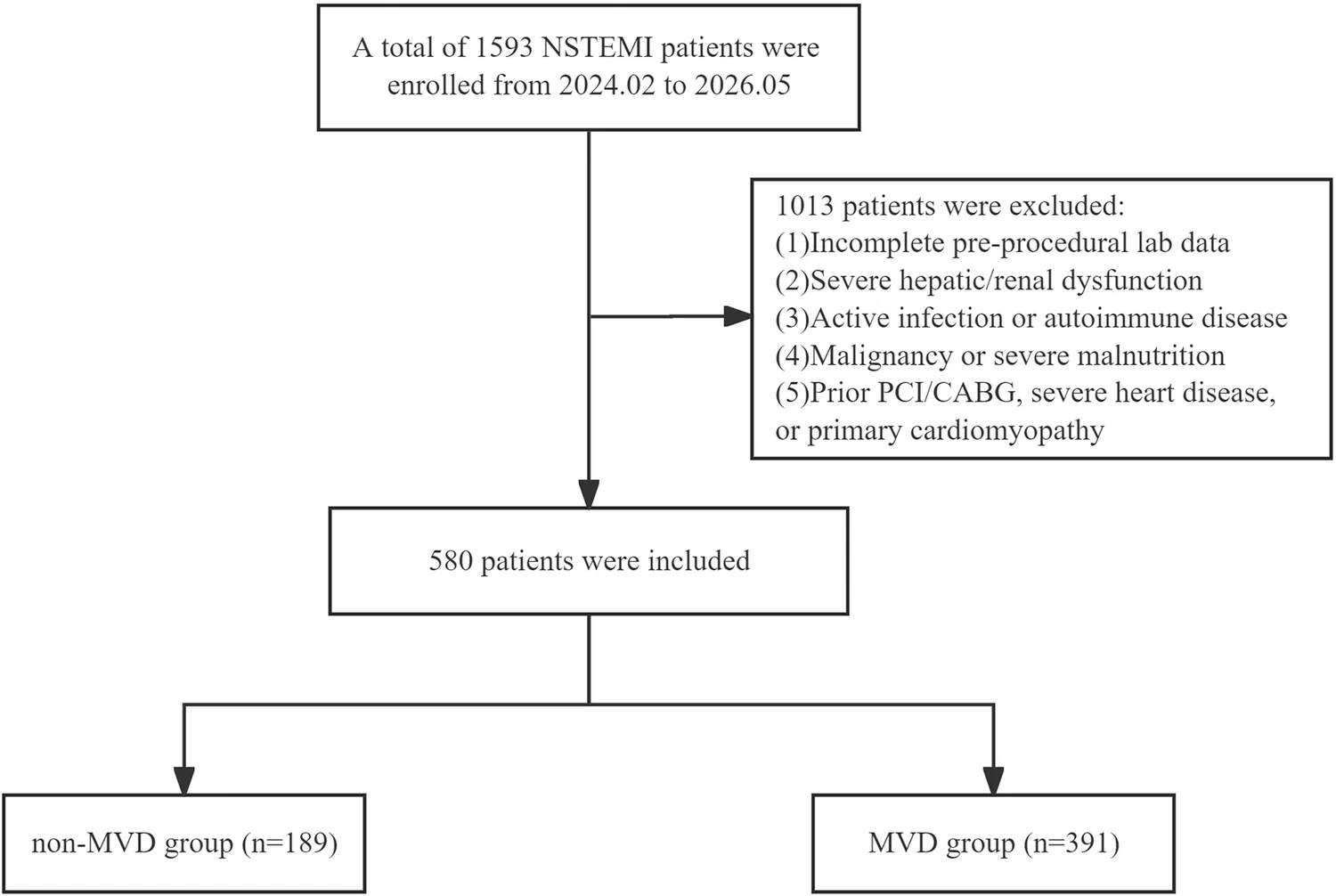
Flowchart of the study population inclusion and exclusion process.

### 2.2 Clinical data collection and definitions

All clinical data were extracted from the hospital's electronic medical record system, and the study reporting adhered to the Strengthening the Reporting of Observational Studies in Epidemiology (STROBE) guidelines (24). The collected data included demographic characteristics, lifestyle factors, medical history, admission medications, vital signs, and a comprehensive panel of laboratory parameters.

#### 2.2.1 Definitions of core biomarkers

The FPR was calculated as fibrinogen (g/L) divided by the platelet count ( ×  10^9^/L).

The ApoB/ApoA1 ratio was calculated as ApoB (g/L) divided by ApoA1 (g/L).

#### 2.2.2 Primary outcome measure

The primary outcome measure of this study was severe MVD, defined as stenosis of the left main coronary artery ≥ 50%; or vascular stenosis ≥ 70% in at least two of the three major territories supplied by the left anterior descending artery, left circumflex artery, and right coronary artery (25).

### 2.3 Statistical analysis

#### 2.3.1 Comparison of baseline characteristics

The Shapiro–Wilk test was used to assess the normality of continuous variables (26). Normally distributed data were described as the mean ± standard deviation, and inter-group comparisons were performed using the independent-samples t-test. Non-normally distributed data were expressed as the median [interquartile range (IQR)], and comparisons between groups were conducted using the Mann–Whitney U test (27). Categorical variables were presented as frequencies (percentages) and compared using Pearson's chi-square test or Fisher's exact test (28). Missing data were handled using multiple imputation by chained equations (MICE) (Supplementary Table S1) (29). Five imputed datasets were generated using predictive mean matching (PMM), with 50 iterations per imputation chain. The primary statistical analyses and model development were conducted using the imputed datasets, whereas baseline characteristics were analyzed using the original data.

#### 2.3.2 Association analysis between biomarkers and lesion severity

The Jonckheere–Terpstra trend test was employed to analyze the trend of the FPR and the ApoB/ApoA1 ratio with the increasing severity of coronary lesions (30). Spearman's rank correlation was utilized to evaluate the correlation strength between these two indicators and lesion severity (31).

#### 2.3.3 Logistic regression and collinearity testing

Three progressively adjusted binary logistic regression models were constructed to assess the independent associations of the biomarkers with severe MVD (32). Model 1 was an unadjusted model; Model 2 was partially adjusted for age, sex, and body mass index (BMI); and Model 3 was further adjusted for smoking status, history of hypertension, history of diabetes, admission heart rate, diastolic blood pressure, low-density lipoprotein cholesterol (LDL-C), N-terminal pro-B-type natriuretic peptide (NT-proBNP), hemoglobin, and lipoprotein(a) [Lp(a)], in addition to the variables included in Model 2. All continuous predictor variables underwent Z-score standardization prior to inclusion in the models. The variance inflation factor (VIF) was calculated to assess multicollinearity within the models (33).

#### 2.3.4 Dose-response relationship

Restricted cubic spline (RCS) models were used to characterize the continuous dose-response relationship between each biomarker and MVD risk (34). The RCS models used the same covariate adjustment strategy as Model 3, with adjustment for all covariates, including smoking status, low-density lipoprotein cholesterol (LDL-C), and lipoprotein(a) [Lp(a)]. In the RCS analyses, four knots were placed at the 5th, 27.5th, 72.5th, and 95th percentiles of the respective biomarker distributions, and the median of each biomarker was set as the reference value for estimating odds ratios (ORs), corresponding to an OR of 1. Wald chi-square tests were used to evaluate the overall and nonlinear associations between each biomarker and MVD risk. *P* values for overall association and nonlinearity were reported separately to assess overall statistical significance and departure from linearity.

#### 2.3.5 Model predictive efficacy and incremental value

Receiver operating characteristic (ROC) curves and the area under the curve (AUC) were used to evaluate model discrimination. DeLong's test was employed to compare the AUCs among different models (35). The continuous net reclassification improvement (NRI) and integrated discrimination improvement (IDI) were calculated to evaluate the incremental predictive value provided by the newly added biomarkers (36).

#### 2.3.6 Sensitivity and anatomical severity analyses

Given that a binary outcome may obscure gradations in the severity of coronary artery disease, a supplementary sensitivity analysis was performed. Based on the number of angiographically involved coronary territories, coronary anatomical disease burden was reclassified into a three-category outcome: ≤1-vessel disease (reference category), 2-vessel disease, and 3-vessel or left main disease. A multinomial logistic regression model was used to assess the independent associations of FPR and the ApoB/ApoA1 ratio with different levels of coronary anatomical disease burden (37). The covariate adjustment strategy for the multinomial model was identical to that used in the fully adjusted model (Model 3) to assess the robustness of the associations of the two biomarkers across levels of coronary disease burden.

#### 2.3.7 Nomogram construction, internal validation, and clinical utility

A visual nomogram was constructed based on the fully adjusted multivariable logistic regression model. Internal validation was performed using 1,000 bootstrap resamples (38), and a calibration curve was generated to evaluate the model's calibration. Decision curve analysis (DCA) was conducted to assess the net clinical benefit of the model across various threshold probabilities (39).

#### 2.3.8 Subgroup and interaction analyses

Subgroup analyses were performed, stratified by age, sex, BMI, hypertension, diabetes, low-density lipoprotein cholesterol (LDL-C), and estimated glomerular filtration rate (eGFR) (40). Within each subgroup, odds ratios (ORs) were estimated after adjustment for all remaining covariates included in the fully adjusted model (Model 3), excluding the stratifying variable itself. For each subgroup variable, effect modification and heterogeneity were assessed by introducing a multiplicative interaction term between the combined biomarker predictor and the subgroup variable into the fully adjusted logistic regression model. *P* values for within-subgroup associations and *P* values for interaction were calculated and reported separately.

All statistical analyses were performed using R software (version 4.5.0). A two-sided *P* < 0.05 was considered statistically significant.

### 2.4 Informed consent and ethical approval

This study was approved by the Medical Ethics Committee of the Affiliated Hospital of Xuzhou Medical University (Approval No.: XYFY2025-KL179-01), and the research protocol strictly adhered to the Declaration of Helsinki (41). As this study was a retrospective medical record analysis without any additional clinical interventions, the ethics committee granted a waiver of written informed consent from the patients. All patient information was anonymized and de-identified, and used solely for scientific research analysis.

## 3 Results

### 3.1 Baseline characteristics

A total of 580 patients with NSTEMI who underwent CAG were included in this study and divided into a non-MVD group (*n* = 189) and an MVD group (*n* = 391) based on the angiographic results (Tables 2, 3). Compared with the non-MVD group, patients in the MVD group were significantly older (median 67.0 vs. 58.0 years, *P* < 0.001) and had higher proportions of comorbid hypertension (62.1% vs. 51.9%, *P* = 0.023) and diabetes (30.2% vs. 21.7%, *P* = 0.041), along with a longer duration of diabetes (*P* = 0.014). However, there were no statistically significant differences between the two groups regarding sex, height, weight, smoking and drinking status, and other comorbidities (all *P* > 0.05).

**Table 2:** Baseline demographics and medical history of the study population.

Characteristics	Non-MVD group (*n* = 189)	MVD group (*n* = 391)	*P* value
Demographics			
Age (years)	58.00 [51.00, 71.00]	67.00 [56.00, 74.00]	<0.001
Gender (Male), *n* (%)	143 (75.7)	290 (74.2)	0.775
Height (cm)	170.00 [160.00, 172.00]	168.00 [160.00, 172.00]	0.461
Weight (kg)	70.00 [63.00, 80.00]	70.00 [63.00, 80.00]	0.842
Vital Signs			
HR (bpm)	70.00 [66.00, 79.00]	73.00 [68.00, 81.00]	0.046
SBP (mmHg)	131.00 [119.00, 144.00]	134.00 [120.00, 145.00]	0.318
DBP (mmHg)	81.00 [75.00, 90.00]	78.00 [71.00, 86.00]	0.010
Medical History & Lifestyle			
Smoking status, *n* (%)			0.592
Never	124 (65.6)	273 (69.8)	
Current	53 (28.0)	96 (24.6)	
Former	12 (6.3)	22 (5.6)	
Drinking status, *n* (%)			0.211
Never	145 (76.7)	321 (82.1)	
Current	40 (21.2)	60 (15.3)	
Former	4 (2.1)	10 (2.6)	
History of hypertension, *n* (%)	98 (51.9)	243 (62.1)	0.023
History of diabetes, *n* (%)	41 (21.7)	118 (30.2)	0.041
Duration of diabetes (years)	0.00 [0.00, 0.00]	0.00 [0.00, 1.00]	0.014
History of stroke, *n* (%)	24 (12.7)	65 (16.6)	0.268
History of dyslipidemia, *n* (%)	1 (0.5)	6 (1.5)	0.436
Family history of CHD, *n* (%)	2 (1.1)	6 (1.5)	1.000
Medications			
Antiplatelet therapy, *n* (%)	67 (35.4)	135 (34.5)	0.900
ACEI/ARB, *n* (%)	41 (21.7)	109 (27.9)	0.135
Beta-blocker, *n* (%)	10 (5.3)	28 (7.2)	0.500
CCB, *n* (%)	40 (21.2)	103 (26.3)	0.210
Diuretics, *n* (%)	15 (7.9)	42 (10.7)	0.360
Statins, *n* (%)	34 (18.0)	79 (20.2)	0.603
Nitrates, *n* (%)	29 (15.3)	59 (15.1)	1.000
Hypoglycemic agents, *n* (%)	29 (15.3)	84 (21.5)	0.101
Insulin, *n* (%)	4 (2.1)	24 (6.1)	0.056

Data are presented as median [IQR], mean ± SD, or number (percentage). HR, heart rate; SBP, systolic blood pressure; DBP, diastolic blood pressure; CHD, coronary heart disease; ACEI, angiotensin-converting enzyme inhibitor; ARB, angiotensin receptor blocker; CCB, calcium channel blocker; MVD, multi-vessel disease.

**Table 3:** Baseline clinical, angiographic, and laboratory characteristics of the study population.

Characteristics	Non-MVD group (*n* = 189)	MVD group (*n* = 391)	*P* value
Angiographic Characteristics			
LM(≥50%), *n* (%)	0 (0.0)	68 (17.4)	<0.001
LAD(≥70%), *n* (%)	112 (59.3)	358 (91.6)	<0.001
LCX (≥70%), *n* (%)	24 (12.7)	319 (81.6)	<0.001
RCA(≥70%), *n* (%)	22 (11.6)	320 (81.8)	<0.001
Laboratory Findings			
WBC ( × 10^9^/L)	7.30 [5.90, 8.60]	7.30 [5.90, 8.70]	0.958
N ( × 10^9^/L)	4.89 [3.77, 6.28]	4.86 [3.73, 6.18]	0.917
L ( × 10^9^/L)	1.70 [1.30, 2.00]	1.60 [1.20, 2.02]	0.306
M ( × 10^9^/L)	0.46 [0.36, 0.57]	0.47 [0.37, 0.61]	0.561
Hb (g/L)	140.09 ± 15.99	135.93 ± 17.46	0.006
RDW (%)	12.80 [12.40, 13.25]	12.80 [12.40, 13.20]	0.584
PLT ( × 10⁹/L)	223.00 [191.00, 274.50]	215.00 [178.00, 258.00]	0.059
MPV (fL)	10.00 [9.40, 10.80]	10.20 [9.57, 11.10]	0.055
FIB (g/L)	2.66 [2.22, 3.24]	3.13 [2.58, 3.78]	<0.001
hs-cTn (ng/L)	183.50 [62.67, 491.00]	249.00 [93.00, 583.00]	0.063
NT-proBNP (pg/mL)	348.50 [104.03, 880.11]	631.50 [262.25, 1,432.75]	<0.001
TC (mmol/L)	4.38 [3.66, 5.10]	4.39 [3.74, 5.24]	0.592
TG (mmol/L)	1.56 [1.04, 2.26]	1.46 [1.09, 2.05]	0.462
HDL-C (mmol/L)	0.91 [0.80, 1.05]	0.91 [0.79, 1.06]	0.634
LDL-C (mmol/L)	2.34 [1.92, 2.84]	2.50 [1.95, 3.04]	0.089
ApoA1 (g/L)	1.19 [1.07, 1.33]	1.17 [1.04, 1.31]	0.169
ApoB (g/L)	0.92 [0.78, 1.08]	0.95 [0.77, 1.14]	0.395
Lp(a) (mg/L)	203.50 [113.00, 358.25]	246.00 [137.50, 455.25]	0.025
AST (U/L)	28.00 [20.75, 40.00]	27.00 [20.00, 47.00]	0.790
ALT (U/L)	21.00 [15.00, 30.50]	21.00 [16.00, 31.00]	0.805
TP (g/L)	67.40 [63.50, 70.95]	66.60 [63.40, 71.00]	0.682
Alb (g/L)	41.50 [39.20, 43.70]	41.20 [38.50, 43.80]	0.608
TBIL (*μ*mol/L)	10.60 [7.30, 14.50]	10.45 [7.60, 14.20]	0.993
Urea (mmol/L)	5.61 [4.71, 6.94]	5.99 [4.79, 7.37]	0.080
SCr (μmol/L)	62.00 [54.50, 74.00]	65.00 [57.00, 76.50]	0.073
UA (μmol/L)	310.00 [250.50, 370.50]	316.00 [257.50, 371.00]	0.494
eGFR (mL/min/1.73m^2^)	120.00 [111.18, 120.00]	120.00 [105.12, 120.00]	0.130
FPG (mmol/L)	5.52 [4.90, 6.60]	5.80 [5.02, 7.47]	0.032

Data are presented as median [IQR], mean ± SD, or number (percentage). LAD, left anterior descending; LCX, left circumflex; RCA, right coronary artery; WBC, white blood cell; Hb, hemoglobin; RDW, red blood cell distribution width; PLT, platelet; MPV, mean platelet volume; FIB, fibrinogen; hs-cTn, high-sensitivity cardiac troponin; NT-proBNP, N-terminal pro-B-type natriuretic peptide; TC, total cholesterol; TG, triglyceride; HDL-C, high-density lipoprotein cholesterol; LDL-C, low-density lipoprotein cholesterol; ApoA1, apolipoprotein A1; ApoB, apolipoprotein B; Lp(a), lipoprotein(a); AST, aspartate aminotransferase; ALT, alanine aminotransferase; TP, total protein; Alb, albumin; TBIL, total bilirubin; SCr, serum creatinine; UA, uric acid; eGFR, estimated glomerular filtration rate; FPG, fasting plasma glucose; MVD, multi-vessel disease.

Regarding vital signs and laboratory findings, the MVD group exhibited a significantly higher resting heart rate (median 73.0 vs. 70.0 bpm, *P* = 0.046) and a significantly lower diastolic blood pressure (median 78.0 vs. 81.0 mmHg, *P* = 0.010). Multiple biomarkers showed significant abnormalities, including significantly elevated levels of NT-proBNP (median 631.50 vs. 348.50 pg/mL, *P* < 0.001), fibrinogen (median 3.13 vs. 2.66 g/L, *P* < 0.001), Lp(a) (median 246.00 vs. 203.50 mg/L, *P* = 0.025), and fasting plasma glucose (FPG) (median 5.80 vs. 5.52 mmol/L, *P* = 0.032), as well as a significant decrease in hemoglobin (Hb) (135.93 ± 17.46 vs. 140.09 ± 15.99 g/L, *P* = 0.006). Apart from a marginally higher proportion of insulin use in the MVD group (6.1% vs. 2.1%, *P* = 0.056), the use rates of other conventional cardiovascular and hypoglycemic medications showed no significant differences between the two groups (all *P* > 0.05).

CAG characteristics further confirmed that atherosclerotic lesions were more extensive and severe in the MVD group: 17.4% of patients in this group had left main (LM) coronary artery stenosis ≥ 50%, whereas no patients in the non-MVD group had LM stenosis ≥ 50% (*P* < 0.001). Additionally, the proportions of stenosis ≥ 70% in the left anterior descending (LAD) artery, left circumflex (LCX) artery, and right coronary artery (RCA) were all significantly higher than those in the non-MVD group (LAD: 91.6% vs. 59.3%; LCX: 81.6% vs. 12.7%; RCA: 81.8% vs. 11.6%, all *P* < 0.001).

### 3.2 Trend and correlation analysis of FPR and the apoB/ApoA1 ratio

Correlation analyses were conducted to quantify the associations between the two residual risk biomarkers and the severity of coronary atherosclerotic lesions. The Jonckheere–Terpstra trend test revealed that, with the increasing severity of coronary lesions, both the FPR (*P* < 0.001) and the ApoB/ApoA1 ratio (*P* = 0.037) exhibited a statistically significant upward trend. Spearman's rank correlation analysis further confirmed that both biomarkers exhibited weak-to-moderate positive correlations with the burden of multivessel stenotic lesions (FPR: r = 0.256, *P* < 0.001; ApoB/ApoA1: r = 0.087, *P* = 0.037; Figure 2), suggesting that these two circulating indicators can be utilized to non-invasively assess the overall burden of coronary atherosclerosis.

**Figure 2:**
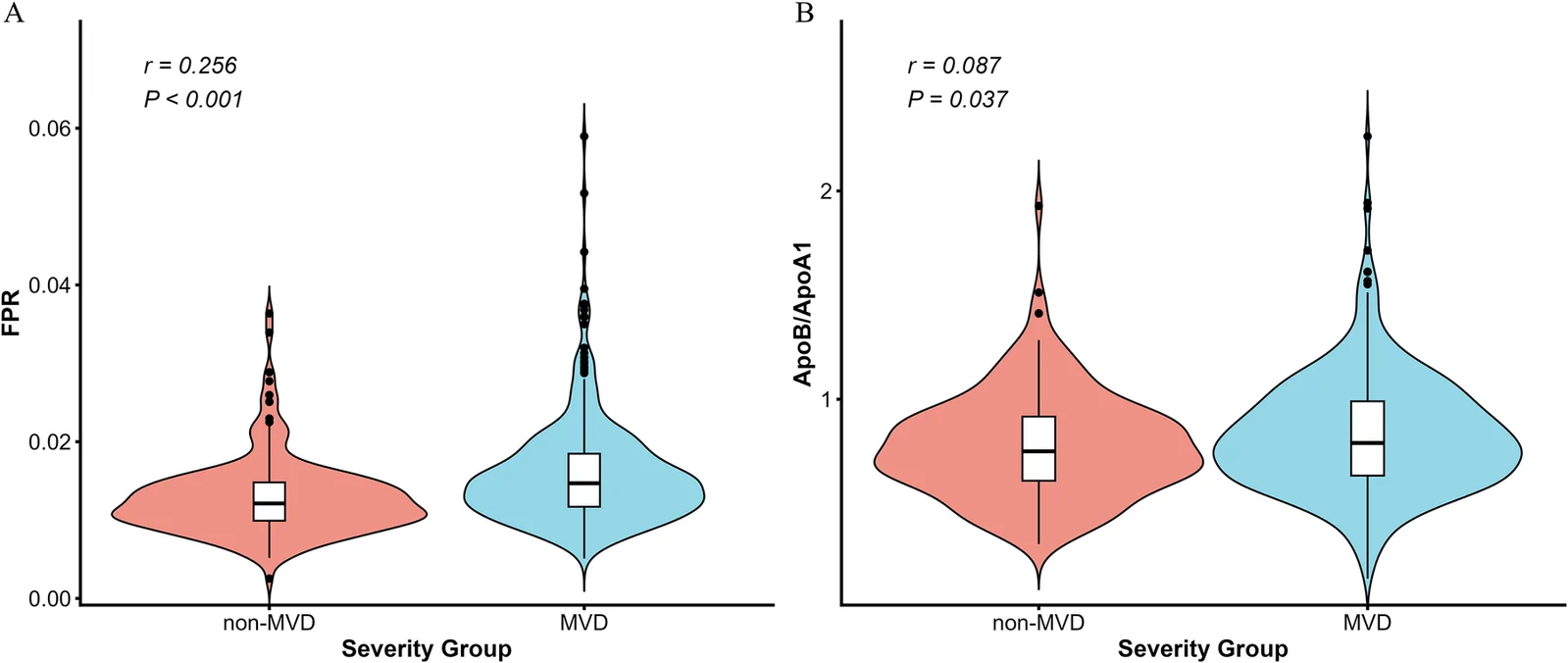
Distribution and correlation of FPR and ApoB/ApoA1 ratio with coronary artery disease severity. Distributions of (**A**) FPR and (**B**) ApoB/ApoA1 ratio in the non-MVD and MVD groups.

### 3.3 Independent predictors of severe MVD

Three progressively adjusted binary logistic regression models were constructed to assess the independent associations of FPR and the ApoB/ApoA1 ratio with severe MVD (Table 4). The crude model (Model 1) showed that an elevated FPR (OR=2.01, 95% CI: 1.58–2.60, *P* < 0.001) and an elevated ApoB/ApoA1 ratio (OR=1.29, 95% CI: 1.07–1.57, *P* = 0.010) were both significantly and positively associated with severe MVD. After partial adjustment for age, sex, and body mass index (BMI) in Model 2, the associations of both biomarkers with severe MVD remained statistically significant (FPR: OR=1.79, *P* < 0.001; ApoB/ApoA1: OR=1.41, *P* < 0.001). In the fully adjusted model (Model 3), after further adjustment for smoking status, history of hypertension, history of diabetes, admission heart rate, diastolic blood pressure, LDL-C, NT-proBNP, hemoglobin, and Lp(a), FPR remained independently associated with severe MVD (OR=1.77, 95% CI: 1.36–2.33, *P* < 0.001), whereas the association of the ApoB/ApoA1 ratio was attenuated and was no longer statistically significant (OR=1.28, 95% CI: 0.97–1.71, *P* = 0.086). All variance inflation factors (VIFs) were <5. Overall, FPR, a marker of residual inflammatory risk, remained independently associated with severe MVD, whereas the ApoB/ApoA1 ratio did not remain independently associated with severe MVD after full adjustment.

**Table 4:** Logistic regression analysis of FPR and apoB/ApoA1 ratio for the risk of MVD.

Variable	Model 1 (Unadjusted)		Model 2 (Partially Adjusted)		Model 3 (Fully Adjusted)	
	OR (95% CI)	*P*-value	OR (95% CI)	*P*-value	OR (95% CI)	*P*-value
FPR	2.01 (1.58–2.60)	<0.001	1.79 (1.41–2.33)	<0.001	1.77 (1.36–2.33)	<0.001
ApoB/ApoA1	1.29 (1.07–1.57)	0.010	1.41 (1.15–1.74)	<0.001	1.28 (0.97–1.71)	0.086

Model 1: Crude model, unadjusted for any potential confounding factors. Model 2: Partially adjusted model, adjusted for age, sex, and body mass index (BMI). Model 3: Fully adjusted model, further adjusted for smoking status, history of hypertension, history of diabetes, admission heart rate, diastolic blood pressure, low-density lipoprotein cholesterol (LDL-C), NT-proBNP, hemoglobin (Hb), and lipoprotein(a) levels based on Model 2. FPR, fibrinogen-to-platelet ratio; ApoB/ApoA1, apolipoprotein B to apolipoprotein A1 ratio; OR, odds ratio; CI, confidence interval; LDL-C, low-density lipoprotein cholesterol.

### 3.4 Dose-response relationships between biomarkers and severe MVD

To further characterize the dose-response relationships of FPR and the ApoB/ApoA1 ratio with severe MVD, restricted cubic spline (RCS) regression models were used to model continuous dose-response curves relating the levels of the two biomarkers to severe MVD status (Figure 3). The overall association tests indicated that higher FPR levels were significantly and positively associated with severe MVD (*P* overall < 0.001), whereas the overall association between the ApoB/ApoA1 ratio and severe MVD did not reach statistical significance (*P* overall = 0.390). Tests for nonlinearity were not statistically significant for either biomarker (FPR: *P* non-linea*r* = 0.081; ApoB/ApoA1: *P* non-linea*r* = 0.759), suggesting an approximately linear, positive association between FPR and severe MVD, whereas no clear dose-response relationship was observed for the ApoB/ApoA1 ratio.

**Figure 3:**
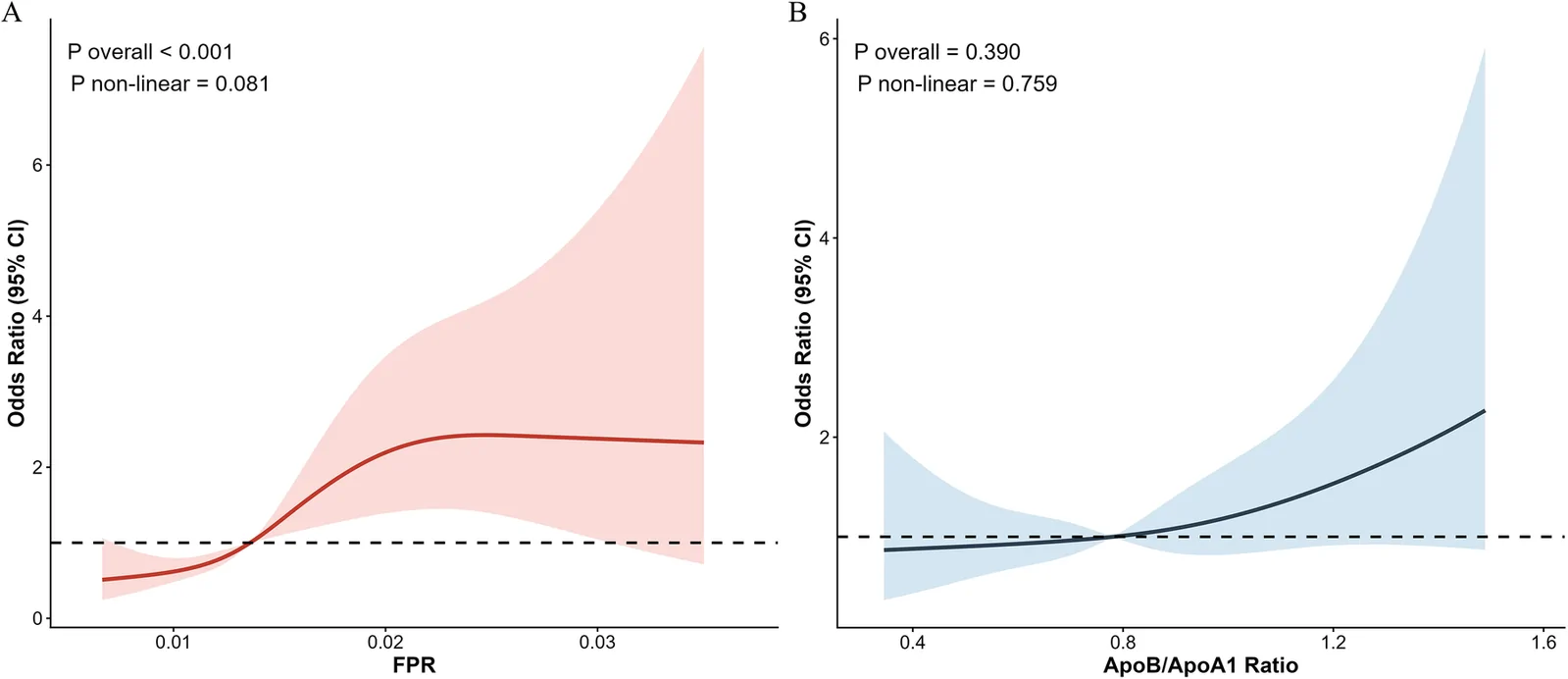
Restricted cubic spline curves for the dose-response relationships with severe multivessel disease risk. (**A**) FPR. (**B**) ApoB/ApoA1 ratio.

### 3.5 Incremental discriminative value of combining FPR and the apoB/ApoA1 ratio

To evaluate the incremental clinical value of the combined assessment of the two biomarkers, receiver operating characteristic (ROC) curves, continuous net reclassification improvement (NRI), and integrated discrimination improvement (IDI) were used to quantify the incremental discriminative value of adding FPR and the ApoB/ApoA1 ratio to the baseline clinical model (Table 5, Figure 4). The baseline model, incorporating only traditional risk factors, exhibited limited discriminatory ability for MVD, with an area under the curve (AUC) of 0.683 (95% CI: 0.637–0.728). After FPR was added to the baseline model, the AUC was 0.714 (DeLong *P* = 0.017), with an NRI of 0.372 and an IDI of 0.031; after the ApoB/ApoA1 ratio was added to the baseline model, the AUC was 0.687 (DeLong *P* = 0.437), with an NRI of 0.179 (*P* = 0.040) and an IDI of 0.005 (*P* = 0.063). When FPR and the ApoB/ApoA1 ratio were simultaneously added to the baseline model, the AUC of the combined biomarker model reached 0.719 (95% CI: 0.676–0.762). DeLong's test indicated a modest but statistically significant improvement in discrimination compared with the baseline model (*P* = 0.008). The combined biomarker model also yielded a continuous NRI of 0.488 (95% CI: 0.326–0.650, *P* < 0.001) and an IDI of 0.037 (95% CI: 0.022–0.052, *P* < 0.001). Compared with the model including FPR alone, the AUC of the combined biomarker model increased from 0.714 to 0.719 (*P* = 0.266); however, the NRI was 0.184 (*P* = 0.034) and the IDI was 0.006 (*P* = 0.046). Compared with the model incorporating fibrinogen and platelet count, the FPR model had an AUC of 0.714 versus 0.709 (DeLong *P* = 0.184), with an NRI of 0.168 (*P* = 0.046) and an IDI of 0.004 (*P* = 0.046).

**Table 5:** Incremental discriminative value of FPR and apoB/ApoA1 ratio for severe MVD.

Model Comparison	Reference Model AUC (95% CI)	New Model AUC (95% CI)	DeLong *P*-value	NRI (95% CI)	NRI *P*-value	IDI (95% CI)	IDI *P*-value
Base+FPR vs. Base	0.683 (0.637–0.728)	0.714 (0.670–0.757)	0.017	0.372 (0.206–0.539)	<0.001	0.031 (0.017–0.045)	<0.001
Base+ApoB/ApoA1 vs. Base	0.683 (0.637–0.728)	0.687 (0.641–0.732)	0.437	0.179 (0.008–0.349)	0.040	0.005 (−0.000–0.011)	0.063
Base+FPR+ApoB/ApoA1 vs. Base	0.683 (0.637–0.728)	0.719 (0.676–0.762)	0.008	0.488 (0.326–0.650)	<0.001	0.037 (0.022–0.052)	<0.001
Base+FPR vs. Base+Fibrinogen+Platelet	0.709 (0.665–0.753)	0.714 (0.670–0.757)	0.184	0.168 (0.003–0.333)	0.046	0.004 (0.000–0.008)	0.046
Base+FPR+ApoB/ApoA1 vs. Base+FPR	0.714 (0.670–0.757)	0.719 (0.676–0.762)	0.266	0.184 (0.014–0.355)	0.034	0.006 (0.000–0.012)	0.046

The base model includes age, sex, body mass index (BMI), smoking status, history of hypertension, history of diabetes, admission heart rate, diastolic blood pressure, low-density lipoprotein cholesterol (LDL-C), NT-proBNP, hemoglobin (Hb), and lipoprotein(a). AUC, area under the curve; CI, confidence interval; NRI, net reclassification improvement; IDI, integrated discrimination improvement; FPR, fibrinogen-to-platelet ratio; ApoB/ApoA1, apolipoprotein B to apolipoprotein A1 ratio; LDL-C, low-density lipoprotein cholesterol.

**Figure 4:**
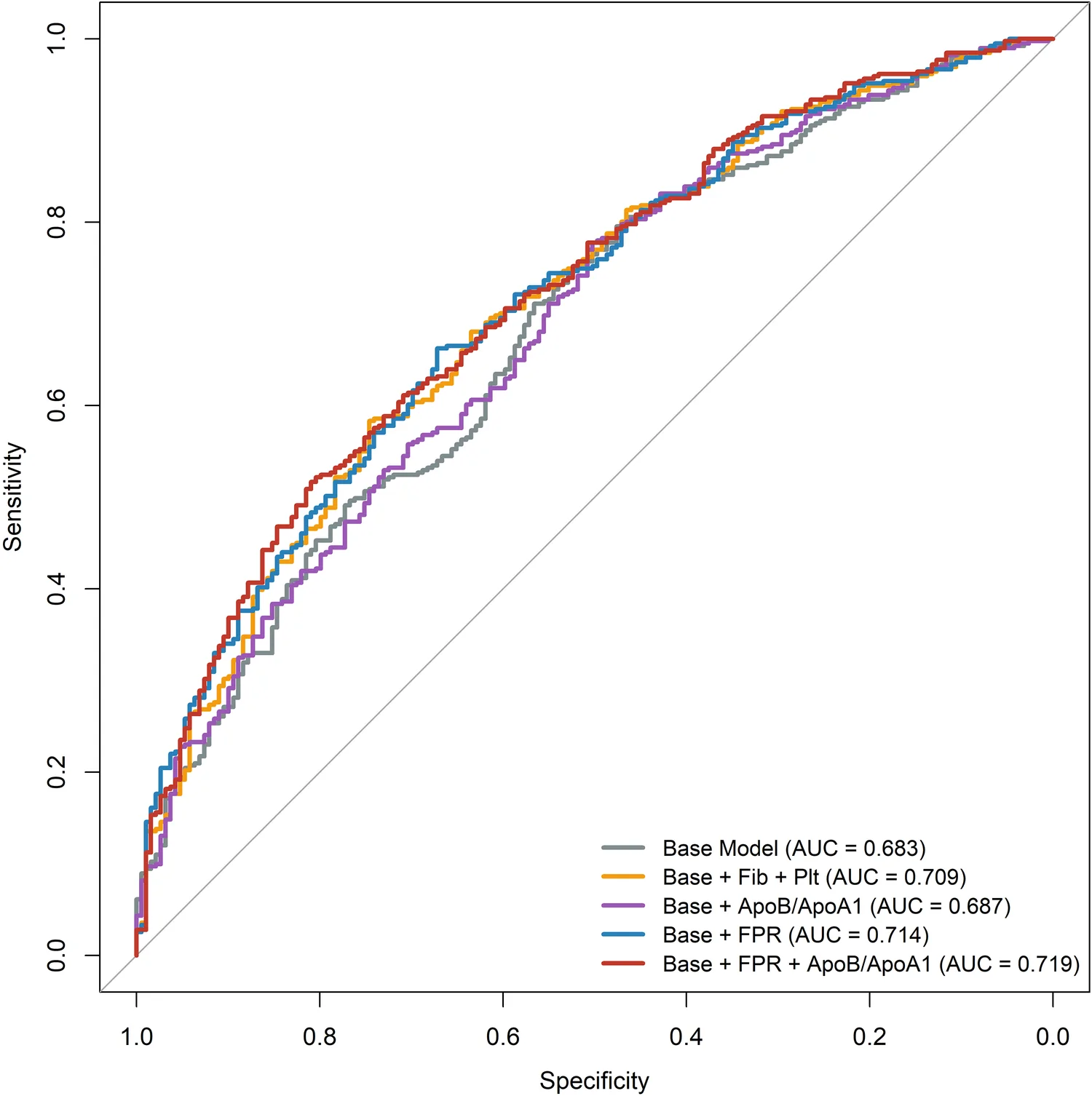
ROC curves of different clinical models for predicting severe multivessel disease.

### 3.6 Sensitivity analysis by coronary anatomical severity

To further evaluate the associations of FPR and the ApoB/ApoA1 ratio with different levels of coronary anatomical disease burden, patients were reclassified according to the number of angiographically involved coronary territories into ≤1-vessel disease, 2-vessel disease, and 3-vessel/Left main (LM) disease, and a sensitivity analysis was performed using multinomial logistic regression (Table 6). In the fully adjusted multinomial model, with ≤1-vessel disease as the reference category, each 1-SD increase in FPR was significantly associated with both 2-vessel disease (OR=1.47, 95% CI: 1.06–2.02, *P* = 0.019) and 3-vessel/LM disease (OR=1.94, 95% CI: 1.46–2.58, *P* < 0.001), and the global test was statistically significant (*P* < 0.001). Each 1-SD increase in the ApoB/ApoA1 ratio was not significantly associated with 2-vessel disease (OR=1.05, 95% CI: 0.74–1.50, *P* = 0.773) but was significantly associated with 3-vessel/LM disease (OR=1.43, 95% CI: 1.06–1.94, *P* = 0.021), with a global *P* value of 0.031. These findings indicated that FPR remained consistently associated with different levels of coronary anatomical disease burden, whereas the association of the ApoB/ApoA1 ratio was primarily evident in the highest anatomical disease-burden category (3-vessel/LM disease), further supporting the robustness of the primary findings.

**Table 6:** Multinomial logistic regression analysis of FPR and apoB/ApoA1 according to coronary anatomical severity.

Variable	2-vessel vs. ≤ 1-vessel OR (95% CI)	*P*-value	3-vessel/LM vs. ≤ 1-vessel OR (95% CI)	*P*-value	Global *P*-value
FPR	1.47 (1.06–2.02)	0.019	1.94 (1.46–2.58)	<0.001	<0.001
ApoB/ApoA1	1.05 (0.74–1.50)	0.773	1.43 (1.06–1.94)	0.021	0.031

The ≤1-vessel group was used as the reference category. The 2-vessel group was defined as involvement of two major coronary territories without left main disease, whereas the 3-vessel/LM group was defined as involvement of all three major coronary territories or left main stenosis ≥50%. FPR and ApoB/ApoA1 were simultaneously included in the multinomial logistic regression model. The model was adjusted for age, sex, body mass index (BMI), smoking status, history of hypertension, history of diabetes, admission heart rate, diastolic blood pressure, low-density lipoprotein cholesterol (LDL-C), NT-proBNP, hemoglobin (Hb), and lipoprotein(a) [Lp(a)]. Continuous variables were standardized, and ORs are presented per 1-SD increase. Global *P*-values were derived from likelihood-ratio tests.

### 3.7 Nomogram construction and clinical utility

Based on the fully adjusted multivariable logistic regression model, clinical variables, FPR, and the ApoB/ApoA1 ratio were integrated to construct a nomogram to assist in identifying severe MVD before coronary angiography (Figure 5). Internal validation was performed using 1,000 bootstrap resamples, and the nomogram yielded a Brier score of 0.1907. The calibration curve (Figure 6A) showed that the model-predicted probabilities were generally consistent with the observed proportions, with a mean absolute error (MAE) of 0.0276 and a maximum absolute error of 0.0440, indicating good internal calibration.

**Figure 5:**
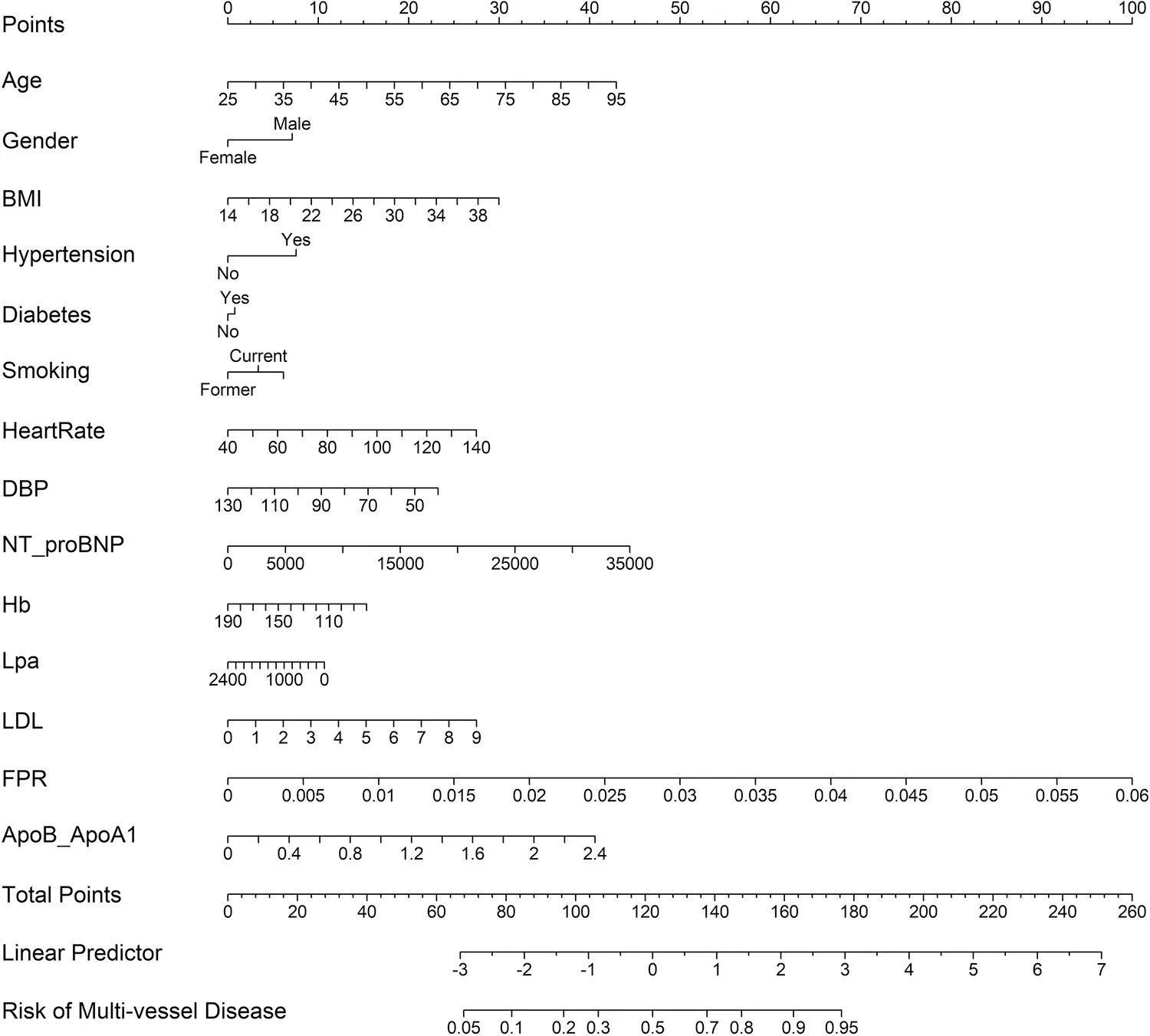
Nomogram for pre-angiographic risk stratification of severe multivessel coronary disease in patients With NSTEMI.

**Figure 6:**
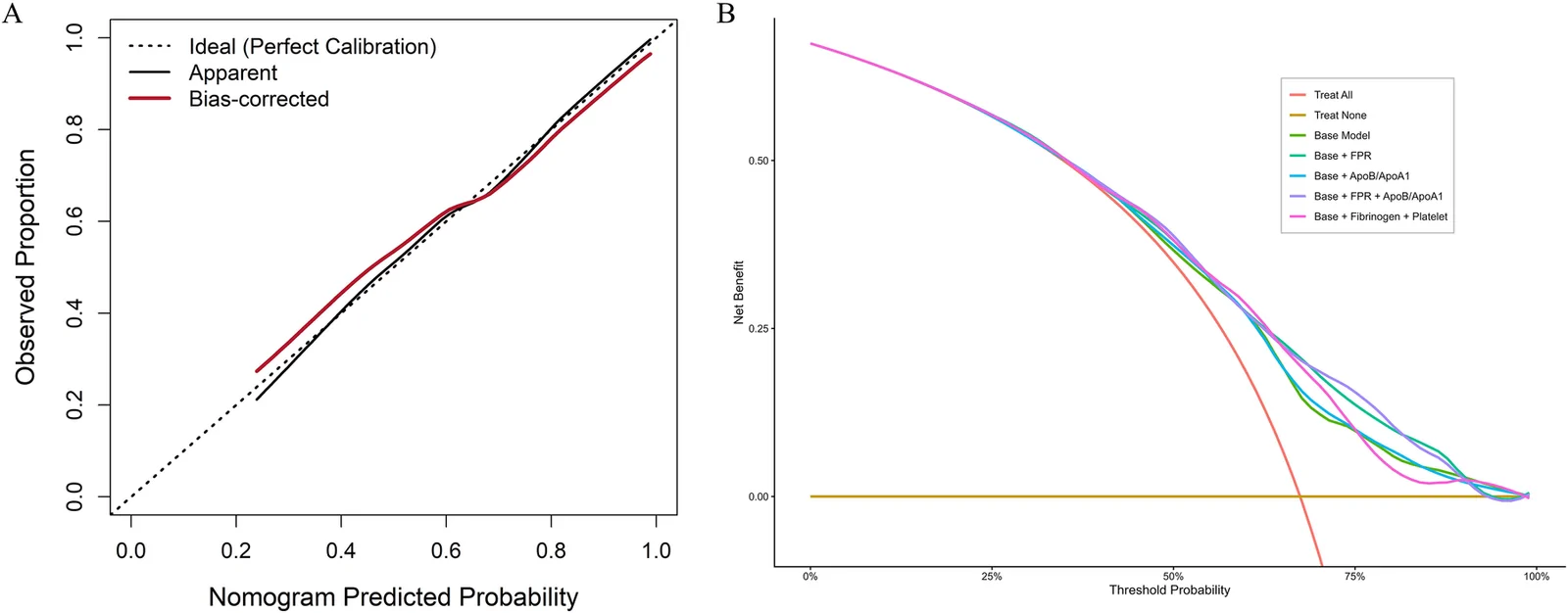
Calibration and decision curve analysis of the nomogram model. (**A**) Calibration plot showing ideal, apparent, and bias-corrected calibration. (**B**) Decision curves comparing five models with the treat-all and treat-none strategies.

Decision curve analysis (DCA; Figure 6B) was used to assess the net benefit of the model for clinical decision-making. Across the evaluated threshold probability range of 0.20–0.80, the dual-biomarker model maintained a positive net benefit and generally yielded greater net benefit than the treat-all and treat-none strategies; compared with the baseline model, its advantage was mainly observed at moderate-to-high threshold probabilities. At representative threshold probabilities, the net benefits of the dual-biomarker and baseline models were 0.5384 and 0.5350, respectively, at Pt = 0.30; 0.3897 and 0.3707, respectively, at Pt = 0.50; and 0.1833 and 0.1075, respectively, at Pt = 0.70. These findings suggest that the nomogram has good internal calibration and provides some additional net benefit at selected moderate-to-high threshold probabilities, supporting its potential use as an adjunctive tool for pre-angiographic risk stratification in patients with NSTEMI scheduled to undergo coronary angiography.

### 3.8 Subgroup and interaction analyses

To examine the consistency of the association between the combined biomarker index (per 1-SD increase) and severe MVD across subgroups, subgroup and interaction analyses were performed across prespecified strata (Figure 7). No significant interactions were observed across strata defined by age (≥65 years: OR=1.90; <65 years: OR=1.90, *P* for interaction = 0.973), sex (male: OR=1.92; female: OR=1.98, *P* for interaction = 0.793), BMI (≥24: OR=1.76; <24: OR=1.90, *P* for interaction = 0.962), history of hypertension (yes: OR=1.59; no: OR=2.19, *P* for interaction = 0.204), eGFR (≥90: OR=1.78; <90: OR=1.46, *P* for interaction = 0.969), or LDL-C (≥1.8: OR=1.77; <1.8: OR=2.99, *P* for interaction = 0.164) (all *P* values for interaction > 0.05), indicating no clear effect modification of the association between the combined biomarker index and severe MVD by these factors.

**Figure 7:**
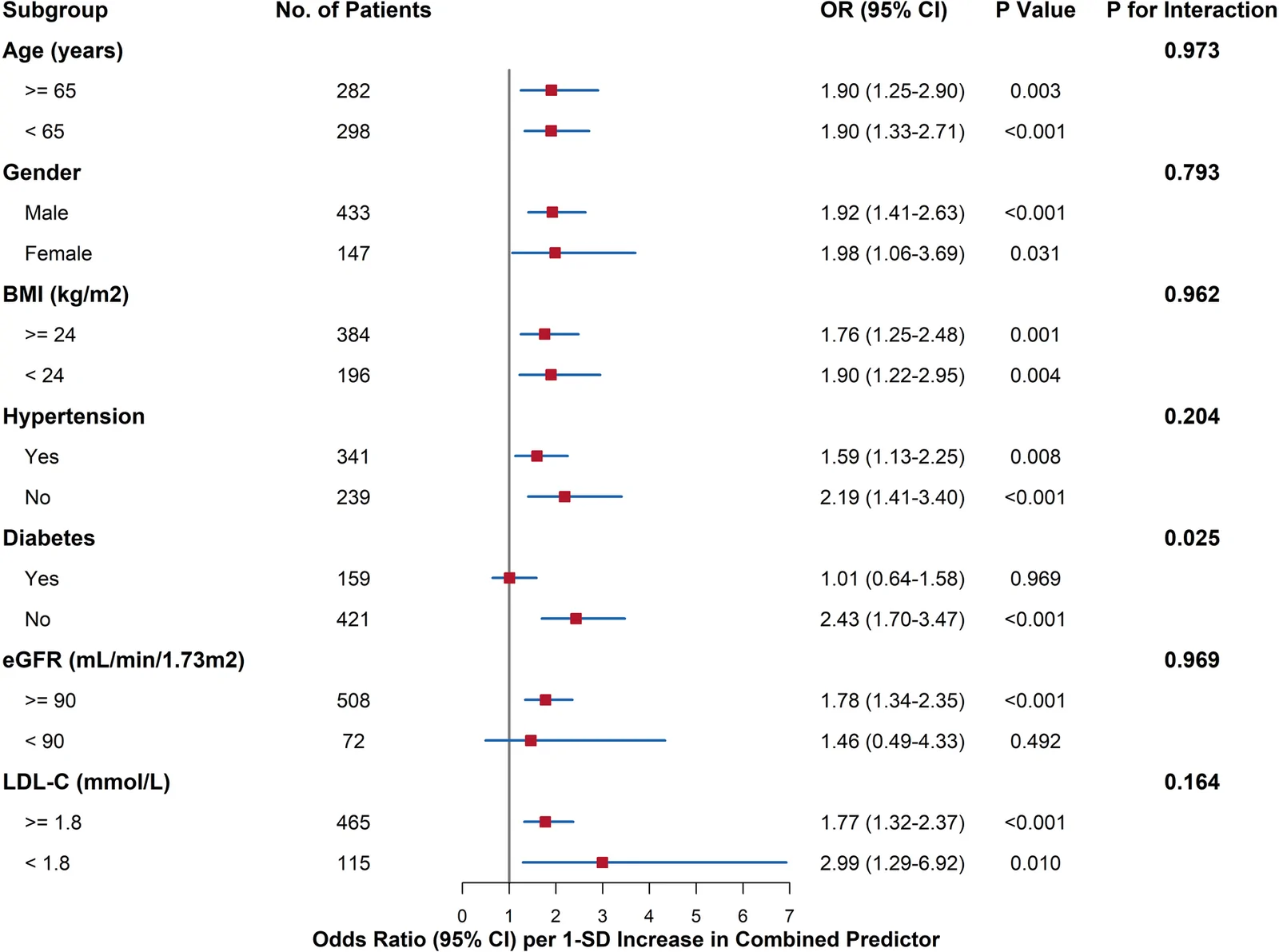
Subgroup analysis of the combined predictor for multivessel disease risk.

Stratification by diabetes status, however, suggested potential effect heterogeneity (*P* for interaction = 0.025). Among patients without diabetes, the combined biomarker index was significantly and positively associated with severe MVD (OR=2.43, 95% CI: 1.70–3.47, *P* < 0.001), whereas among patients with diabetes, this association was attenuated and did not reach statistical significance (OR=1.01, 95% CI: 0.64–1.58, *P* = 0.969). These findings suggest that diabetes status may modify the association between the combined biomarker index and severe MVD. However, given the relatively small sample size of the diabetes subgroup (*n* = 159), this finding should be further validated in larger samples and independent external cohorts.

## 4 Discussion

### 4.1 Principal study findings

Based on cross-sectional coronary angiographic data from 580 patients with NSTEMI, this study found that FPR remained independently and positively associated with severe multivessel coronary disease (MVD), with an approximately linear dose-response relationship; in contrast, the association of the ApoB/ApoA1 ratio was attenuated and did not reach statistical significance after full adjustment for conventional risk factors, although the sensitivity analysis showed that the ApoB/ApoA1 ratio remained significantly associated with 3-vessel/left main disease, representing the highest anatomical disease burden. Adding FPR, a marker of residual inflammatory risk, to the clinical baseline model significantly improved discrimination. Further addition of the ApoB/ApoA1 ratio, reflecting residual lipid risk, did not significantly increase the AUC but provided additional value in risk reclassification and discrimination, as indicated by the NRI and IDI. Although the AUC did not differ significantly between the FPR model and the model incorporating fibrinogen and platelet count, both the NRI and IDI indicated statistically significant improvements, suggesting that the incremental value of FPR may be more evident in individual risk reclassification and improved separation of predicted probabilities than in a marked increase in overall AUC. The nomogram constructed using these indicators showed good calibration after internal validation by bootstrap resampling and provided some additional net benefit at selected moderate-to-high threshold probabilities. Subgroup analyses suggested that diabetes status may modify the association between the combined biomarker index and severe MVD. Taken together, these findings support the potential utility of an integrated assessment of residual inflammatory and lipid risks as an adjunctive approach for pre-angiographic risk stratification of coronary anatomical disease burden in patients with NSTEMI.

### 4.2 Potential mechanisms linking FPR to residual inflammatory risk

In the era of intensive lipid-lowering therapy, residual low-grade chronic inflammation is a core driving factor for the continuous progression of atherosclerosis and the widespread involvement of plaques across multiple vessels (42). Fibrinogen possesses dual pro-inflammatory and pro-coagulant effects: on the one hand, it upregulates endothelial adhesion molecules to promote monocyte infiltration into plaques; on the other hand, it serves as a coagulation substrate to accelerate microthrombus formation, thereby exacerbating vascular endothelial injury (43, 44). Platelets serve as a bridge between inflammation and thrombosis; upon activation, they release copious amounts of inflammatory mediators, amplifying the local vascular inflammatory response (45, 46). By integrating fibrinogen and platelet count into a single ratio, FPR simultaneously captures inflammation- and coagulation-related information, may reflect an integrated state of inflammatory and coagulation activation, and serves as a composite surrogate marker of residual inflammatory risk (47). In this study, FPR levels were significantly higher in the MVD group and remained independently associated with MVD after adjustment for traditional risk factors and other inflammatory and biochemical indicators. In the sensitivity analysis, FPR also remained significantly associated with 2-vessel and 3-vessel/left main disease, suggesting that FPR may reflect a state of inflammation-coagulation activation associated with extensive coronary atherosclerosis and acute ischemic events.

### 4.3 Potential mechanisms linking the apoB/ApoA1 ratio to residual lipid homeostatic imbalance

Even when LDL-C targets are achieved, the substantial accumulation of pro-atherogenic lipoprotein particles *in vivo* still confers a residual lipid risk (48). ApoB is present in all pro-atherogenic lipoproteins, enabling the precise quantification of the total burden of pathogenic particles (49). ApoA1 is the core structural protein of high-density lipoprotein (HDL), mediating reverse cholesterol transport, exerting antioxidant effects, and protecting the vascular endothelium (50, 51). An elevated ApoB/ApoA1 ratio indicates that the pro-atherogenic lipid effects have overwhelmed the vascular protective mechanisms, resulting in an imbalance of lipid metabolic homeostasis (52–54). Prolonged lipid imbalance causes extensive lipid deposition across multiple vessels, promoting foam cell formation and the expansion of the necrotic core within plaques, ultimately culminating in diffuse, severe multivessel stenosis (55, 56). The present findings suggest that the residual lipid risk reflected by the ApoB/ApoA1 ratio may not be equally evident across all levels of coronary disease burden, but may be more apparent in patients with the highest anatomical disease burden and in individual risk reclassification. These findings suggest that, beyond traditional risk factors and information related to inflammation and coagulation, the imbalance between atherogenic lipoprotein burden and protective lipoproteins may provide a distinct additional dimension of risk information for complex coronary disease, thereby highlighting its potential complementary value within an inflammation-lipid dual-axis risk assessment framework.

### 4.4 Potential complementary value of combined assessment of the inflammation–lipid dual axis

Most existing studies have solely explored the association of either FPR or the ApoB/ApoA1 ratio with coronary lesion severity; few have combined the two for pre-angiographic identification of severe MVD in patients with NSTEMI (57). Furthermore, previous risk models have predominantly focused on clinical endpoints such as long-term mortality and myocardial infarction, lacking dedicated assessment tools specifically targeting the complexity of coronary anatomy (58).

At the molecular pathophysiological level, inflammatory and lipid pathways may interact bidirectionally through multiple mechanisms (59). The ApoB/ApoA1 imbalance leads to the massive retention of pro-atherogenic lipoproteins within the vascular wall, which are then attacked by local reactive oxygen species (ROS) and converted into oxidized low-density lipoprotein (oxLDL) possessing potent pro-inflammatory activity (60, 61). oxLDL not only induces endothelial cells to express adhesion molecules for monocyte recruitment but also prompts macrophages to engulf lipids in an unregulated manner, forming foam cells and releasing pro-inflammatory cytokines such as interleukin (IL)-1β and IL-6 (62). These inflammatory factors, in turn, activate oxidase systems within endothelial cells and macrophages, generating more ROS to accelerate lipid oxidation. This forms a pathological closed loop of “lipid accumulation–oxidative modification–inflammatory infiltration” (63–65).

During this process, the inflammatory-coagulation and nutritional acute-phase response reflected by FPR may further amplify local inflammatory reactions within the plaque. Fibrinogen may participate in macrophage inflammatory activation through receptor-mediated pathways involving Mac-1 (CD11b/CD18) (66). Simultaneously, oxLDL can induce platelet activation or pre-activation via receptors such as CD36 and LOX-1, prompting the release of chemokines such as CXCL4/PF4. Through pathways involving macrophages, endothelial cells, and matrix metalloproteinases (MMPs), these processes may promote extracellular matrix degradation, necrotic core formation and expansion, and plaque destabilization, thereby contributing to alterations in the plaque microenvironment (67, 68).

Therefore, a single biomarker can only capture the risk of a single pathway, resulting in assessment blind spots. By integrating biomarkers reflecting the complementary inflammatory and lipid pathways, the combined assessment further improved risk reclassification for severe MVD beyond that achieved by individual biomarkers or the baseline clinical model and yielded some additional net benefit on decision curve analysis. The present findings do not support a synergistic interpretation in which the two biomarkers jointly produce a significant additional improvement in overall discrimination; rather, FPR appears to be the principal source of incremental discrimination, whereas the ApoB/ApoA1 ratio may provide limited complementary reclassification information. Taken together, these findings suggest that the two biomarkers may capture complementary aspects of residual inflammatory and lipid risk, thereby providing more comprehensive biological information for pre-angiographic risk stratification of severe coronary disease.

### 4.5 Potential explanations for effect heterogeneity in the diabetes subgroup

The present findings suggest potential effect modification by diabetes status: the combined biomarker index remained significantly and positively associated with severe MVD in patients without diabetes, whereas the association was attenuated in patients with diabetes. This heterogeneity can be explained from the perspective of metabolic disturbances: diabetic patients suffer from multiple systemic metabolic disorders, concomitant with persistent low-grade inflammation, dyslipidemia, insulin resistance, and extensive vascular endothelial damage. In a hyperglycemic environment, multiple pathways involving inflammation, lipids, and oxidative stress are simultaneously hyperactivated (69). Against this complex metabolic background, FPR and the ApoB/ApoA1 ratio may reflect only a subset of the inflammatory, coagulation-related, and lipid abnormalities, and the discriminative information they provide regarding coronary anatomical disease may be influenced by other metabolic pathways. These findings suggest that diabetes-specific models incorporating glycated hemoglobin (HbA1c) and markers of insulin resistance may be needed for patients with NSTEMI and diabetes, rather than direct application of the present dual-axis assessment framework. Such models may provide a more refined basis for stratified risk assessment. However, the diabetes subgroup included only 159 patients, resulting in limited statistical power, and the possibility of a false-positive finding cannot be excluded. Future studies with larger sample sizes, prespecified stratification by diabetes status, and independent validation cohorts are therefore needed to confirm this exploratory finding.

### 4.6 Translational clinical utility of the dual-biomarker nomogram

Pre-Angiographic Adjunctive Risk Stratification to Facilitate Diagnostic and Procedural Preparation. The nomogram uses clinical data collected at admission and routine blood-based laboratory parameters to provide additional information on coronary anatomical disease burden before invasive assessment. For patients identified pre-angiographically as having a higher likelihood of severe MVD, the model may facilitate early recognition of the possibility of complex coronary anatomy and inform Heart Team discussions, preprocedural planning for complete revascularization, and preparation of intravascular imaging equipment, including IVUS and OCT systems, potentially reducing the need for *ad hoc* intraprocedural strategy changes and shortening clinical decision-making time (70, 71).

Support for Revascularization Decision-Making and Procedural Planning. Pre-angiographic estimation of the likelihood of severe MVD may provide useful contextual information before definitive anatomical assessment. Patients at high risk, highly suggestive of LM or severe multivessel stenosis, can be prioritized for evaluating the benefits of surgical bypass; conversely, low-risk populations with single-vessel disease can undergo streamlined intervention processes, facilitating the rational allocation of catheterization laboratory resources (72). Through these objective, quantitative indicators, clinicians can communicate more intuitively and professionally with patients and their families preoperatively, vastly improving the efficiency of shared medical decision-making. Simultaneously, this strategy helps avoid inadequate interventional procedures in patients with complex anatomical lesions, thereby significantly improving long-term vascular patency rates and prognostic outcomes.

Guidance for individualized secondary prevention interventions. Based on the levels of the FPR and the ApoB/ApoA1 ratio, the dominant type of residual risk can be differentiated: For patients predominantly exhibiting an elevated FPR, anti-inflammatory therapies can be intensified; for those predominantly showing an elevated ApoB/ApoA1 ratio, novel potent lipid-lowering drugs can be initiated (25, 73). This achieves targeted intervention and breaks through the traditional “one-size-fits-all” treatment paradigm. This dual-axis phenotyping strategy provides clear biological monitoring targets for long-term follow-up after discharge, enabling physicians to flexibly implement personalized, targeted interventions. Compared to traditional standardized treatment models, this pathway not only enhances the efficacy of pharmacological therapies but also holds promise for reducing the recurrence risk of long-term cardiovascular events.

Health economic advantages. Both the FPR and the ApoB/ApoA1 ratio are derived from routine admission complete blood counts, coagulation profiles, and lipid panels, without incurring any additional testing costs for patients, fully embodying the low-cost and high-return characteristics of their clinical application. Against the backdrop of current healthcare payment reforms and the pooling of medical resources, this zero-additional-cost, non-invasive predictive tool possesses immense promotional value, being particularly suited for early risk identification in primary healthcare institutions. It not only alleviates the financial burden on patients but also effectively utilizes existing medical resources, facilitating the promotion of a tiered healthcare delivery system regionally and elevating the overall quality of care for complex cardiovascular patients.

### 4.7 Limitations and future research directions

Although this study explored the value of assessing residual inflammatory and lipid risks for adjunctive pre-angiographic risk stratification of severe MVD in patients with NSTEMI, several important limitations should be acknowledged. Firstly, this study utilized a single-center, retrospective design and lacked an independent external cohort for cross-validation, which may introduce selection bias to some extent and thus limit the generalizability of the findings. However, as a major cardiovascular disease diagnosis and treatment center in Northern Jiangsu, our center's patient sources broadly cover Jiangsu, Shandong, Anhui, and surrounding areas. The admitted cases exhibit good continuity and heterogeneity, largely reflecting the clinical characteristics of real-world NSTEMI patients. Even so, multicenter, prospective studies incorporating independent external validation cohorts are required to verify the robustness of these results.

Secondly, this study focused on pre-angiographic cross-sectional anatomical risk anticipation and has not yet established a long-term follow-up mechanism to include data on long-term major adverse cardiovascular events (MACE). Therefore, the direct explanatory and predictive capability of this “inflammation-lipid” dual-axis model regarding the long-term adverse prognosis of NSTEMI patients remains to be elucidated in future longitudinal follow-up studies.

In addition, all participants were hospitalized patients with NSTEMI who had been clinically selected to undergo coronary angiography; consequently, the study population was generally at relatively high risk of coronary artery disease and may have been subject to spectrum bias. Therefore, the findings cannot be directly generalized to general outpatient populations or low-risk patients presenting with chest pain. Moreover, patients with a history of PCI or CABG were excluded because prior revascularization may substantially alter native coronary anatomy, while factors such as long-term antithrombotic therapy may affect coagulation and platelet status, thereby complicating the interpretation of FPR levels and coronary anatomical disease burden. Although this exclusion criterion helped maintain the homogeneity of the study endpoint, it may also have reduced the representativeness of the study sample relative to the broader real-world NSTEMI population.

Overall, despite these limitations, the findings support FPR as an important adjunctive biomarker reflecting residual inflammatory risk and severe coronary disease burden, whereas the ApoB/ApoA1 ratio may provide complementary information regarding residual lipid risk. Combined assessment of the two biomarkers may capture different pathological dimensions of coronary risk in patients with NSTEMI and provide additional clinical information to support pre-angiographic identification and risk stratification of severe MVD in patients undergoing invasive evaluation.

## 5 Conclusion

Based on a systematic analysis of 580 patients with NSTEMI who underwent coronary angiography, this study showed that the combined assessment of the fibrinogen-to-platelet ratio (FPR) and the apolipoprotein B/A1 ratio (ApoB/ApoA1) may aid in identifying severe multivessel coronary disease (MVD) by capturing information across the two dimensions of residual inflammatory and lipid risk. FPR was independently associated with MVD and exhibited an approximately linear dose-response relationship with disease risk. In contrast, the association of the ApoB/ApoA1 ratio was attenuated and was no longer statistically significant after full adjustment, although it retained complementary value in patients with the highest coronary anatomical disease burden and in risk reclassification.

Compared with the baseline model relying solely on traditional risk factors, the combined biomarker model further improved discrimination, with the AUC increasing from 0.683 to 0.719, and yielded significant improvements in risk reclassification (continuous NRI = 0.488) and discrimination (IDI = 0.037). The resulting nomogram showed good internal calibration and provided additional net benefit at selected moderate-to-high threshold probabilities. The findings also suggested potential effect heterogeneity according to diabetes status: the association between the combined biomarker index and severe MVD was more pronounced in patients without diabetes but was markedly attenuated in those with diabetes, suggesting that differences in metabolic background may influence the risk information conveyed by the inflammation–lipid dual-axis assessment.

In summary, the nomogram, which incorporates FPR and the ApoB/ApoA1 ratio together with routinely available admission variables, provides a practical adjunctive tool for pre-angiographic risk stratification in patients with NSTEMI. By capturing complementary information from pathways related to inflammation-coagulation and lipid metabolism, this framework may complement traditional risk-factor assessment, aid in the pre-angiographic identification of patients with a higher likelihood of severe coronary disease, and provide objective information to support the Heart Team in preprocedural assessment, diagnostic and procedural preparation, and revascularization planning, thereby supporting its potential clinical utility.

## Data Availability

The raw data supporting the conclusions of this article will be made available by the authors, without undue reservation.
