# Supplementary material for: Combined assessment of fibrinogen-to-platelet ratio and ApoB/ApoA1 ratio for pre-angiographic risk stratification of severe multivessel coronary disease in NSTEMI
Source: Front Cardiovasc Med. 2026 Oct 5;13:1931950. doi: 10.3389/fcvm.2026.1931950 (PMC13677501; doi:10.3389/fcvm.2026.1931950)
Supplement: Supplementary file 1 [file Table1.pdf]

Supporting materials:

Table S1. Missing data summary of baseline clinical and laboratory variables.

Variables	Missing Count (n)	Missing Rate (%)
Urine protein	88	15.17
NT-proBNP (pg/mL)	63	10.86
FPG (mmol/L)	40	6.90
eGFR (mL/min/1.73m²)	37	6.38
hs-cTn (ng/L)	11	1.90
Urea (mmol/L)	6	1.03
UA (μmol/L)	6	1.03
SCr (μmol/L)	6	1.03
TBIL (μmol/L)	5	0.86
SBP (mmHg)	4	0.69
Alb (g/L)	4	0.69
TP (g/L)	4	0.69
DBP (mmHg)	4	0.69
GGT (U/L)	4	0.69
ALT (U/L)	4	0.69
ALP (U/L)	4	0.69
AST (U/L)	3	0.52

Notes: This table only lists baseline variables with missing values; variables not listed had complete data (0% missing). Missing data were handled using multiple imputation by chained equations (MICE) with the predictive mean matching (PMM) algorithm, generating five imputed datasets with 50 iterations per chain. Abbreviations: NT-proBNP: N-terminal pro-B-type natriuretic peptide; FPG: fasting plasma glucose; eGFR: estimated glomerular filtration rate; hs-cTn: high-sensitivity cardiac troponin; UA: uric acid; SCr: serum creatinine; TBIL: total bilirubin; SBP: systolic blood pressure; Alb: albumin; TP: total protein; DBP: diastolic blood pressure; GGT: gamma-glutamyl transferase; ALT: alanine aminotransferase; ALP: alkaline phosphatase; AST: aspartate aminotransferase.
